# Supplementary material for: Extrafine HFA-beclomethasone-formoterol vs. nonextrafine combination of an inhaled corticosteroid and a long acting β2-agonist in patients with persistent asthma: A systematic review and meta-analysis
Source: PLoS One. 2021 Sep 3;16(9):e0257075. doi: 10.1371/journal.pone.0257075 (PMC8415610; doi:10.1371/journal.pone.0257075)
Supplement: S1 File — (DOCX) [file pone.0257075.s001.docx]

**S1 File. Search strategy used to identify trials**

1. **PubMed**

**Beclometasone**

((((((((((((((((((((((((((((((((((((((((((Beclomethasone[Title/Abstract]) OR Beclometasone[Title/Abstract]) OR Asmabec Clickhaler[Title/Abstract]) OR Ascocortonyl[Title/Abstract]) OR Beclamet[Title/Abstract]) OR Beclo Asma[Title/Abstract]) OR Beclo AZU[Title/Abstract]) OR Beclocort[Title/Abstract]) OR Beclomet[Title/Abstract]) OR Bemedrex Easyhaler[Title/Abstract]) OR Beclomethasone Dipropionate[Title/Abstract]) OR Dipropionate, Beclomethasone[Title/Abstract]) OR Beclorhinol[Title/Abstract]) OR Sanasthmax[Title/Abstract]) OR Becloturmant[Title/Abstract]) OR Beclovent[Title/Abstract]) OR Beconase[Title/Abstract]) OR Becloforte[Title/Abstract]) OR Becodisk[Title/Abstract]) OR Becodisks[Title/Abstract]) OR Propaderm[Title/Abstract]) OR Becotide[Title/Abstract]) OR Sanasthmyl[Title/Abstract]) OR Beconase AQ[Title/Abstract]) OR Bronchocort[Title/Abstract]) OR Junik[Title/Abstract]) OR Qvar[Title/Abstract]) OR Ecobec[Title/Abstract]) OR Beclazone[Title/Abstract]) OR Beclazone Easy Breathe[Title/Abstract]) OR Ventolair[Title/Abstract]) OR Prolair[Title/Abstract]) OR Filair[Title/Abstract]) OR Filair Forte[Title/Abstract]) OR AeroBec Forte[Title/Abstract]) OR Aerobec[Title/Abstract]) OR Nasobec Aqueous[Title/Abstract]) OR Respocort[Title/Abstract]) OR Vancenase[Title/Abstract]) OR Vanceril[Title/Abstract]) OR Aldecin[Title/Abstract]) OR Viarin[Title/Abstract]) OR Apo-Beclomethasone[Title/Abstract]

**Asthma**

(((asthma[Title/Abstract]) OR Asthmas[Title/Abstract]) OR Bronchial Asthma[Title/Abstract]) OR Asthma, Bronchial[Title/Abstract]

**RCTs**

((((((((randomized controlled trial[Publication Type]) OR controlled clinical trial[Publication Type]) OR randomized[Title/Abstract]) OR placebo[Title/Abstract]) OR clinical trials[MeSH Major Topic]) OR randomly[Title/Abstract]) OR trial[Title])) NOT ((animals[MeSH Terms]) NOT humans[MeSH Terms])

1. **Embase**

**Beclometasone**

'beclomethasone':ti,ab

**Asthma**

'Asthma':ti,ab OR 'allergic asthma':ti,ab OR 'aspirin exacerbated respiratory disease':ti,ab OR 'asthmatic state':ti,ab OR 'exercise induced asthma':ti,ab OR 'experimental asthma':ti,ab OR 'extrinsic asthma':ti,ab OR 'intrinsic asthma':ti,ab OR 'mild intermittent asthma':ti,ab OR 'mild OR persistent asthma':ti,ab OR 'moderate persistent asthma':ti,ab OR 'nocturnal asthma':ti,ab OR 'occupational asthma':ti,ab OR 'severe persistent asthma':ti,ab

**RCTs**

'randomized controlled trial'/exp

1. **CENTRAL**

**Beclometasone**

MeSH descriptor: [Beclomethasone] explode all trees OR (Beclomethasone):ti,ab,kw

**Asthma**

MeSH descriptor: [Asthma] explode all trees OR (Asthma):ti,ab,kw OR (Asthma, Aspirin-Induced):ti,ab,kw OR (Asthma, Exercise-Induced):ti,ab,kw OR (Asthma, Occupational):ti,ab,kw OR (Status Asthmaticus):ti,ab,kw
