# Supplementary material for: Extrafine HFA-beclomethasone-formoterol vs. nonextrafine combination of an inhaled corticosteroid and a long acting β2-agonist in patients with persistent asthma: A systematic review and meta-analysis
Source: PLoS One. 2021 Sep 3;16(9):e0257075. doi: 10.1371/journal.pone.0257075 (PMC8415610; doi:10.1371/journal.pone.0257075)
Supplement: S2 File — (DOCX) [file pone.0257075.s002.docx]

**S2 File. References to studies excluded from this review after full-text articles assessed for eligibility.**

**Not randomized controlled trial (n=13)**

1. Ederle K, Multicentre Study Group. Improved control of asthma symptoms with a reduced dose of HFA-BDP extrafine aerosol: an open-label, randomised study. Eur Rev Med Pharmacol Sci. 2003;7(2):45-55.

2. Price D, Martin RJ, Barnes N, Dorinsky P, Israel E, Roche N, et al. Prescribing practices and asthma control with hydrofluoroalkane-beclomethasone and fluticasone: a real-world observational study. J Allergy Clin Immunol. 2010;126(3):511-8.e1-10. doi: 10.1016/j.jaci.2010.06.040.

3. Barnes N, Price D, Colice G, Chisholm A, Dorinsky P, Hillyer EV, et al. Asthma control with extrafine-particle hydrofluoroalkane-beclometasone vs. large-particle chlorofluorocarbon-beclometasone: a real-world observational study. Clin Exp Allergy. 2011;41(11):1521-32. doi: 10.1111/j.1365-2222.2011.03820.x.

4. Brusselle G, Peché R, Van den Brande P, Verhulst A, Hollanders W, Bruhwyler J. Real-life effectiveness of extrafine beclometasone dipropionate/formoterol in adults with persistent asthma according to smoking status. Respir Med. 2012;106(6):811-9. doi: 10.1016/j.rmed.2012.01.010.

5. Popov TA, Petrova D, Kralimarkova TZ, Ivanov Y, Popova T, Peneva M, et al. Real life clinical study design supporting the effectiveness of extra-fine inhaled beclomethasone/formoterol at the level of small airways of asthmatics. Pulm Pharmacol Ther. 2013;26(6):624-9. doi: 10.1016/j.pupt.2013.06.002.

6. Price D, Small I, Haughney J, Ryan D, Gruffydd-Jones K, Lavorini F, et al. Clinical and cost effectiveness of switching asthma patients from fluticasone-salmeterol to extra-fine particle beclometasone-formoterol: a retrospective matched observational study of real-world patients. Prim Care Respir J. 2013;22(4):439-48. doi: 10.4104/pcrj.2013.00088.

7. Price D, Thomas M, Haughney J, Lewis RA, Burden A, von Ziegenweidt J, et al. Real-life comparison of beclometasone dipropionate as an extrafine- or larger-particle formulation for asthma. Respir Med. 2013;107(7):987-1000. doi: 10.1016/j.rmed.2013.03.009.

8. Kuna P, Kupryś-Lipińska I, Dębowski T. Control of asthma in adults treated with beclomethasone and formoterol in extrafine particle formulation in a real-life setting in Poland: the CASPER noninterventional, observational trial. Pol Arch Med Wewn. 2015;125(10):731-40. doi: 10.20452/pamw.3111.

9. Dobbelaere S, Joos G. Inhaled corticosteroids in combination with formoterol as a maintenance and reliever therapy in mild to moderate persistent asthma. Ned Tijdschr Geneeskd. 2016;72(4):276-81. doi: 10.2143/TVG.72.04.2002067.

10. Marth K, Spinola M, Kisiel J, Woergetter C, Petrovic M, Pohl W. Treatment response according to small airway phenotypes: a real-life observational study. Ther Adv Respir Dis. 2016;10(3):200-10. doi: 10.1177/1753465816642635.

11. Dhillon S. Extrafine beclometasone dipropionate/formoterol fumarate metered-dose and dry-powder inhalers in asthma and chronic obstructive pulmonary disease: a profile of their use. Drugs & Therapy Perspectives. 2017;33(6):260-71. doi: 10.1007/s40267-017-0397-7.

12. Ghorani V, Marefati N, Shakeri F, Rezaee R, Boskabady M, Boskabady MH. The Effects of Allium Cepa Extract on Tracheal Responsiveness, Lung Inflammatory Cells and Phospholipase A2 Level in Asthmatic Rats. Iran J Allergy Asthma Immunol. 2018;17(3):221-231.

13. Tamási L, Szilasi M, Gálffy G. Clinical Effectiveness of Budesonide/Formoterol Fumarate Easyhaler® for Patients with Poorly Controlled Obstructive Airway Disease: a Real-World Study of Patient-Reported Outcomes. Adv Ther. 2018;35(8):1140-1152. doi: 10.1007/s12325-018-0753-6.

**No eligible outcomes or data (n=1)**

1. Wolthers OD. Short-term growth and adrenal function in children with asthma treated with inhaled beclomethasone dipropionate hydrofluoroalkane-134a. Pediatr Allergy Immunol. 2006;17(8):613-9. doi: 10.1111/j.1399-3038.2006.00460.x.

**Extrafine treatment in both interventions (n=3)**

1. Harrison LI, Kurup S, Wagner C, Ekholm BP, Larson JS, Kaiser HB. Pharmacokinetics of beclomethasone 17-monopropionate from a beclomethasone dipropionate extrafine aerosol in adults with asthma. Eur J Clin Pharmacol. 2002;58(3):197-201. doi: 10.1007/s00228-002-0466-1.

2. Fardon TC, Burns P, Barnes ML, Lipworth BJ. A comparison of 2 extrafine hydrofluoroalkane-134a-beclomethasone formulations on methacholine hyperresponsiveness. Ann Allergy Asthma Immunol. 2006;96(3):422-30. doi: 10.1016/S1081-1206(10)60909-X.

3. Singh D, van den Berg F, Leaker B, Corradi M, Jabbal S, Collarini S, et al. Comparison of the effect of beclometasone/formoterol in asthma patients after methacholine-induced bronchoconstriction: A noninferiority study using metered dose vs. dry powder inhaler. Br J Clin Pharmacol. 2019;85(4):729-736. doi: 10.1111/bcp.13847.

**Single inhaler extrafine triple therapy (n=1)**

1. Virchow JC, Kuna P, Paggiaro P, Papi A, Singh D, Corre S, et al. Single inhaler extrafine triple therapy in uncontrolled asthma (TRIMARAN and TRIGGER): two double-blind, parallel-group, randomised, controlled phase 3 trials. Lancet. 2019;394(10210):1737-1749. doi: 10.1016/S0140-6736(19)32215-9.

**Single dose effect (n=3)**

1. Rosati P, Porzsolt F. A practical educational tool for teaching child-care hospital professionals attending evidence-based practice courses for continuing medical education to appraise internal validity in systematic reviews. J Eval Clin Pract. 2013;19(4):648-52. doi: 10.1111/j.1365-2753.2012.01889.x.

2. Kuna P, Govoni M, Lucci G, Scuri M, Acerbi D, Stelmach I. Pharmacokinetics and pharmacodynamics of an extrafine fixed pMDI combination of beclometasone dipropionate/formoterol fumarate in adolescent asthma. Br J Clin Pharmacol. 2015;80(3):569-80. doi: 10.1111/bcp.12640.

3. Boer S, Honkoop PJ, Loijmans RJB, Snoeck-Stroband JB, Assendelft WJJ, Schermer TRJ, et al. Personalised exhaled nitric oxygen fraction (FENO)-driven asthma management in primary care: a FENO subgroup analysis of the ACCURATE trial. ERJ Open Res. 2020;6(3):00351-2019. doi: 10.1183/23120541.00351-2019.

**Acute effects (n=4)**

1. Ayres JG, Simmons JL, Stampone P. Acute safety of beclomethasone dipropionate in a new CFC-free propellant system in asthmatic patients. Respir Med. 1999;93(1):27-32. doi: 10.1016/s0954-6111(99)90073-2.

2. Martinez FD, Chinchilli VM, Morgan WJ, Boehmer SJ, Lemanske RF Jr, Mauger DT, et al. Use of beclomethasone dipropionate as rescue treatment for children with mild persistent asthma (TREXA): a randomised, double-blind, placebo-controlled trial. Lancet. 2011;377(9766):650-7. doi: 10.1016/S0140-6736(10)62145-9.

3. O'Connor BJ, Collarini S, Poli G, Brindicci C, Spinola M, Acerbi D, et al. Rapid effects of extrafine beclomethasone dipropionate/formoterol fixed combination inhaler on airway inflammation and bronchoconstriction in asthma: a randomised controlled trial. BMC Pulm Med. 2011;11:60. doi: 10.1186/1471-2466-11-60.

4. Papi A, Corradi M, Pigeon-Francisco C, Baronio R, Siergiejko Z, Petruzzelli S, et al. Beclometasone-formoterol as maintenance and reliever treatment in patients with asthma: a double-blind, randomised controlled trial. Lancet Respir Med. 2013;1(1):23-31. doi: 10.1016/S2213-2600(13)70012-2.
