# Supplementary figures and images for: Extrafine HFA-beclomethasone-formoterol vs. nonextrafine combination of an inhaled corticosteroid and a long acting β2-agonist in patients with persistent asthma: A systematic review and meta-analysis
Source: PLoS One. 2021 Sep 3;16(9):e0257075. doi: 10.1371/journal.pone.0257075 (PMC8415610; doi:10.1371/journal.pone.0257075)

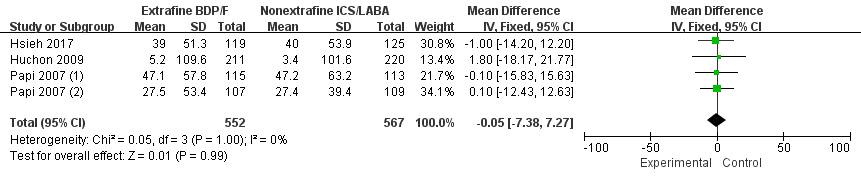

Supplement: S1 Fig — (TIF) [file pone.0257075.s003.tif]

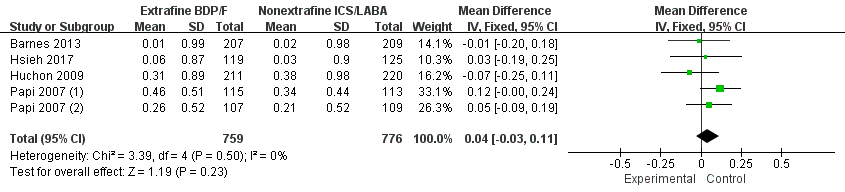

Supplement: S2 Fig — (TIF) [file pone.0257075.s004.tif]

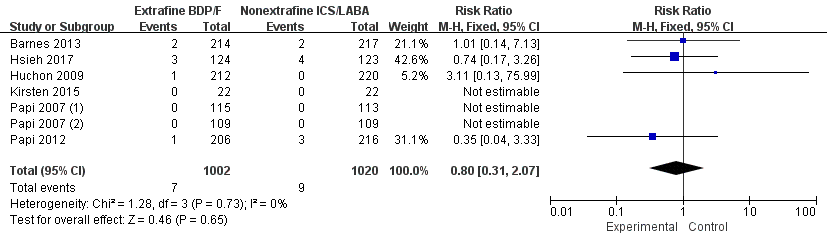

Supplement: S3 Fig — (TIF) [file pone.0257075.s005.tif]

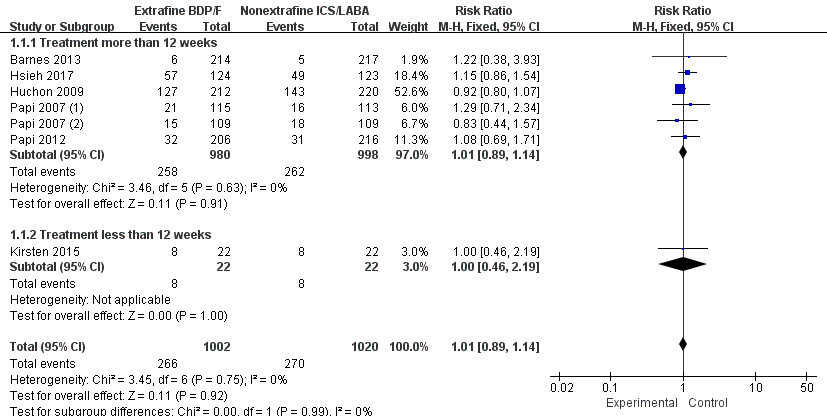

Supplement: S4 Fig — (TIF) [file pone.0257075.s006.tif]

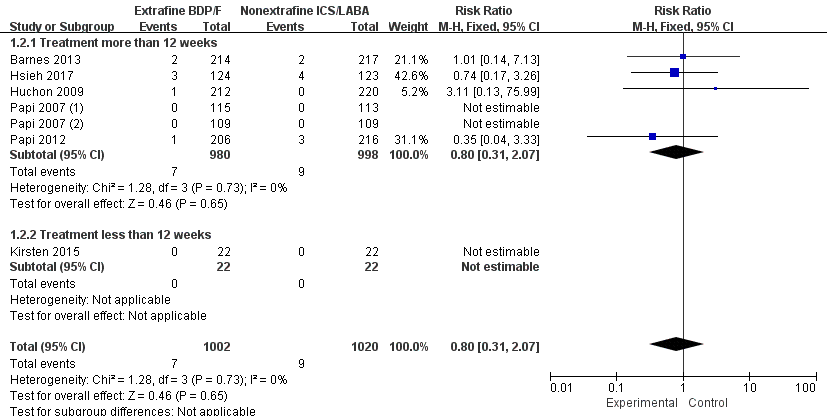

Supplement: S5 Fig — (TIF) [file pone.0257075.s007.tif]
