# Supplementary material for: Extrafine HFA-beclomethasone-formoterol vs. nonextrafine combination of an inhaled corticosteroid and a long acting β2-agonist in patients with persistent asthma: A systematic review and meta-analysis
Source: PLoS One. 2021 Sep 3;16(9):e0257075. doi: 10.1371/journal.pone.0257075 (PMC8415610; doi:10.1371/journal.pone.0257075)
Supplement: Attachment — Submitted filename: Response to Reviewers.docx [file pone.0257075.s009.docx]

Dear editor,

Thank you for your letter and for the reviewers’ comments concerning our manuscript entitled “Extrafine HFA-beclomethasone-formoterol vs nonextrafine combination of an inhaled corticosteroid and a long acting β2-agonist in patients with persistent asthma: a systematic review and meta-analysis” (ID: PONE-D-20-35965). Those comments are valuable and very helpful for revising and improving our paper. We have carefully revised the manuscript according to the reviewers' comments, and the amendments are highlighted in yellow in the revised manuscript. Point by point responses to the reviewers’ comments are as flowing.

**Responses to editor**

**1.** I suggest sub-group analyses to see the impact of dose (200/12ug bid versus 100/6ug bid) and the duration of therapy on outcomes. Lower doses and shorted durations may have reduced the rate of adverse events as example.

**Response:** Dear editor, thank you very much for your kind help! Your suggestion is valuable and very helpful to us. Sub-group analyses were considered when we extracted the data of these trials, however, it’s a pity we did not show them in the manuscript because of the limited number of studies, for example, there was only two small-sample studies (Bulac 2015 and Kirsten 2015) of low dose (200/12ug bid) and short duration (3-4 weeks). According to your suggestion, we have added the sub-group analyses of AE and SAE in the S4 and S5 Figs. (Line 262, 474-477)

**2.** Include the funding source behind the RCTs you included in the meta-analysis.

**Response:** Thank you for your reminder. We have added the funding sources in Table 1. (Page 10)

**3.** It is customary to associate the quantitative results of a meta-analysis with a classification of clinical evidence and the strength of recommendations, such as via the GRADE assessment in meta-analyses, particularly as authors did not perform a trial sequential analysis in the meta-analysis.

**Response:** According to the GRADE assessment system, RCT is of the highest quality level based on the research design. All we have enrolled were RCTs and we made a strict quality assessment according to the entry of PRISMA 2009 checklist which was included in the S1 Table and Fig 2. (Risk of bias summary). We considered the quality of these RCTs were relatively high except for the inevitable unblinding of MDI and DPI. (Line 478)

**4.** I did not find the registration number of this meta-analysis in a prospective register (as PROSPERO) in order to follow the pre-planned protocol.

**Response:** Thank you very much for your kind reminder. We have made a protocol for the meta-analysis, but we did not register in PROSPERO because of its website maintenance when we did this research. We have resubmit the protocol to PROSPERO.

**5.** Please elaborate on why only including RCTs, when only less than 10 studies were found in the literature and these are mostly not recent.

**Response:** We only included RCTs because of their highest quality, in order to ensure the high quality of our data analysis. We searched several databases and the results recently, and these studies were indeed all RCT studies. Our research team has also carried out an RCT of BDP-F vs BUD-F, but the results have not been published.

**6.** Please include a list of excluded full text articles as appendix, including reasons for exclusion.

**Response:** We have made a more detailed description of the key studies excluded, which was shown in S2 File. ( Line 173-174, 467)

**7.** What were the serious adverse events that you pool analyzed?

**Response:** An SAE is not always the same in different studies, most of which is considered to meet any one of the following criteria: is fatal or life-threatening; results in persistent or significant disability/incapacity; constitutes a congenital anomaly/birth defect; requires inpatient hospitalization or prolongation of existing hospitalization; is medically significant, i.e. defined as an event that jeopardizes the patient or may require medical or surgical intervention.

**Journal Requirements**

**1.** Please ensure that your manuscript meets PLOS ONE's style requirements, including those for file naming. The PLOS ONE style templates can be found at

https://journals.plos.org/plosone/s/file?id=wjVg/PLOSOne_formatting_sample_main_body.pdf

**Response:** We have revised the manuscript to meets PLOS ONE's style requirements.

**2.** Please include your tables as part of your main manuscript and remove the individual files. Please note that supplementary tables (should remain/ be uploaded) as separate "supporting information" files.

**Response:** We have included our tables as part of our main manuscript and remove the individual files.

**Responses to** **reviewer #1**

**1.** The protocol of the study is not part of the appendix. Has it been published anywhere? Is it possible to include the protocol in the revision to check for adherence to the protocol and to ensure study can be replicated. The manuscript does not report on any protocol deviation.

**Response:** Thank you very much for your kind help and careful review. We have drafted a protocol of this study and it has not been published anywhere. Recently, we have submitted the protocol to register on PROSPERO.

**2.** The key words on the cover page (4 of them) are more than the key words listed in the search strategy. Is there any reason that key words used in the search are only 2? If there were more key words, please consider including them in the methods. How were you able to ensure that all the relevant articles were included with using only the 2 keys words? From fig 1 flow chart, 929 TOTAL articles were identified from the 3 databases. Is it possible to provide details about the total number of articles identified in each data base after key word search and how many were excluded after the review of the titles of these studies?

**Response:** Thank you for your valuable advice on the revision of the flow chart. We tried to avoid omissions of key studies when we made the search strategies, thus, the key words used in the search were only 2, not including “RCT”. The studies not comparing beclomethasone-formoterol with ICS-LABA or not extrafine particles would be excluded, as was shown in the flow chart. We have made a more detailed description of the studies included and excluded, including the total number of articles identified in each data base after key word search and the number of articles excluded after the review of the titles. Some data were shown in S2 File. ( Line 173-174, 467)

**3.** Line 45 of the abstract results "didn't"- Please consider "did not"

**Response:** We have revised the words in the resubmitted manuscript.( Line 45)

**4.** Line 74 of the introduction: "These years, various drug formulations and inhalation techniques have been developed..." - It is not very clear what these years mean?

**Response:** The first ICS-LABA was developed in 2001, thus "these years" means about twenty years. We have added the detailed year information in the resubmitted manuscript. (Line 73)

**5.** Line 80 of the introduction "pressurized metered dose inhaler (pMDI) " -Consider using only pMDI as the abbreviation has been defined above already.

**Response:** We have revised the words in the resubmitted manuscript. Thank you for your careful review. (Line 80)

**6.** Line 83 of introduction "to generate" -Please consider another word. For the context of delivering medication to a site of action, "generate" may not be the most appropriate word to use.

**Response:** We have replaced "generate" with "improve". (Line 82)

**7.** Line 126 of the methods "and other outcome variables of" - Please check the font size of these words. They are smaller.

**Response:** We have revised it and checked the font size of the whole manuscript to ensure to meet the journal's style requirements. (Line 126)

**8.** Line 147 & 148 of methods "The assessment of risk of biases was performed based on the methodology reported by each enrolled study following the rules specified in the Cochrane Handbook " - Why should the risk of biases be based on the methodology of each enrolled study when only RCTs were included? It may be good to elaborate a little bit more.

**Response:** The risk of biases have been assessed and shown in detail in Fig 2. We included only RCTs in order to ensure the quality of evidence.

**9.** Line 155 of the statistical analysis "group27 " - Please check if it is a spelling mistake.

**Response:** We have revised it and checked the whole manuscript to avoid a spelling mistake. Thank you for your reminder. (Line 155)

**10.** Line 164 of the statistical analysis "We failed to evaluate publication bias with a funnel plot and Egger’s test, due to the limited number of included studies. "- Should the inability to check for publication bias not be included as a limitation of the study?

**Response:** We have added it in the part of limitation in our resubmitted manuscript. (Line 329-331)

**11.** Line 213 of the results outcomes "bodyplethysmography" - Please check the spelling.

**Response:** We have revised it in the resubmitted manuscript. (Line 222)

**12.** Discussion section: How will the fewer number of studies and by extension relatively lower number of patients affect the results?

**Response:** The fewer number of included studies and patients could directly affect the statistical significance of pooled results and also indirectly affect the clinical significance due to the limited population. The number of studies and patients in our study could make a review and pooled meta-analyses but could not have an absolute conclusion on some outcomes.

**13.** References section: Line 346, reference 1- Please consider including the date you accessed this reference via the website.

**Response:** We have added the date in the resubmitted manuscript. (Line 349)

**14.** Appendix: Titles of figures 3,4,& 5: These titles need to be revised slightly. From the way it is written, the respective outcomes have effects on the treatment.

**Response:** We have revised it in the resubmitted manuscript. (Line 468-473)

**Other changes:**

**1.** “Extrafine HFA-beclomethasone-formoterol vs nonextrafine combination of an inhaled corticosteroid and a long acting β2-agonist in patients with persistent asthma: a systematic review and meta-analysis” were corrected as “Extrafine HFA-beclomethasone-formoterol vs. nonextrafine combination of an inhaled corticosteroid and a long acting β2-agonist in patients with persistent asthma: a systematic review and meta-analysis”.(Line 1)

**2.** “Extrafine HFA-beclomethasone-formoterol vs. nonextrafine combination in asthma” were corrected as “Extrafine HFA-beclomethasone-formoterol *vs*. nonextrafine combination in asthma” .(Line 15)

**3.** “Among current therapies, beclometasone dipropionate-formoterol fumarate (BDP-F) fixed combination delivers extrafine particles” were corrected as “Among current therapies, BDP-F fixed combination delivers extrafine particles ”(Line 59)

**4.** “The last search was performed on October 2020.” were corrected as “The last search was performed on June 2021.”(Line 113)

**5.** “In *Bulac 2015*” were corrected as “In Bulac 2015”(Line 230)

**6.** “As we know, this was the first review and meta-analysis comparing extrafine beclometasone dipropionate-formoterol fumarate (BDP-F) fixed combination via pMDI with other ICS-LABAs.” were corrected as “As we know, this was the first review and meta-analysis comparing extrafine BDP-F fixed combination via pMDI with other ICS-LABAs.”(Line 269)

We hope that the revised version of the manuscript is now acceptable for publication in your journal. If there is anything else we should do, please do not hesitate to let us know.

I look forward to hearing from you soon.

Thank you very much.

Yours sincerely,

Chuntao Liu
