# Supplementary material for: Extrafine HFA-beclomethasone-formoterol vs. nonextrafine combination of an inhaled corticosteroid and a long acting β2-agonist in patients with persistent asthma: A systematic review and meta-analysis
Source: PLoS One. 2021 Sep 3;16(9):e0257075. doi: 10.1371/journal.pone.0257075 (PMC8415610; doi:10.1371/journal.pone.0257075)
Supplement: Attachment — Submitted filename: Response to Reviewers.docx [file pone.0257075.s010.docx]

Dear editor,

Thank you for your comments concerning our manuscript entitled “Extrafine HFA-beclomethasone-formoterol vs. nonextrafine combination of an inhaled corticosteroid and a long acting β2-agonist in patients with persistent asthma: a systematic review and meta-analysis” (ID: PONE-D-20-35965R1). Those comments are valuable and very helpful for revising and improving our paper. We have carefully revised the manuscript according to your comments, and the amendments are highlighted in yellow in the revised manuscript. Point by point responses to your comments are as flowing.

**Responses to editor**

**1.** Please follow up on your addition about the results of the subgroup analysis you provided with details of the intended comparisons in the Methods part of the manuscript. Currently, I do not believe the Methods of the manuscript are including any of the subgroup analysis plans.

**Response:** Dear editor, thank you for your reminder. According to your suggestion, we have added the subgroup analysis plans in the part of Methods. (Line 172-173)

**2.** Also, please elaborate on the lack of the GRADE assessment in the limitation of the manuscript, including the justification.

**Response:** Thank you very much for your kind help! We have added the GRADE assessment in the Table 2. (Line 155-157, 229, 230, 273, 274, 283, 284, Page 16-17)

**3.** Similarly, please elaborate in the Discussion part of the manuscript about the two issues of (i) what are the serious adverse drug reactions and why not identifying them, and (ii) why only including RCTs in the analysis and the potential associated risk of bias.

**Response:** Thank you for your valuable advice. We think the elaborate of two issues can be placed in the method section and we add this in the Method part of manuscript. (Line 121, 122, 135-138)

**Journal Requirements**

**1.** Please review your reference list to ensure that it is complete and correct. If you have cited papers that have been retracted, please include the rationale for doing so in the manuscript text, or remove these references and replace them with relevant current references. Any changes to the reference list should be mentioned in the rebuttal letter that accompanies your revised manuscript. If you need to cite a retracted article, indicate the article’s retracted status in the References list and also include a citation and full reference for the retraction notice.

**Response:** We have reviewed our reference list and ensured all reference were complete and correct.

**Other changes:**

**1.** “the clinical effects of extrafine HFA-beclomethasone-formoterol (BDP-F) versus equipotent doses of nonextrafine combination of an inhaled corticosteroid and a long acting β2-agonist (ICS-LABA) in asthma.” were corrected as “the clinical effects of extrafine hydrofluoroalkane (HFA)-beclomethasone-formoterol (BDP-F) versus equipotent doses of nonextrafine combination of an inhaled corticosteroid and a long acting β2-agonist (ICS-LABA) in asthma.”.(Line 27)

**2.** “no significant benefit of extrafine HFA-BDP-F over nonextrafine ICS-LABA in improving Asthma Control Test score (*p* = 0.30)” were corrected as “no significant benefit of extrafine HFA-BDP-F over nonextrafine ICS-LABA in improving Asthma Control Test (ACT) score (*p* = 0.30) ” .(Line 44)

**3.** “the first FDCs containing extrafine formulation of BDP and formoterol (BDP-F) both in the pMDI and dry powder inhaler (DPI) versions” were corrected as “the first FDCs containing extrafine formulation of BDP and BDP-F both in the pMDI and dry powder inhaler (DPI) versions ”(Line 80)

**4.** “compared to those of fluticasone-salmeterol (FP-S) DPI and budesonide-formoterol (BUD-F) DPI” were corrected as “compared to those of FP-S DPI and BUD-F DPI.”(Line 85)

**5.** “a significant decrease in FeNO levels were observed in extrafine BDP-F” were corrected as “a significant decrease in fractional exhaled nitric oxide (FeNO) levels were observed in extrafine BDP-F”(Line 95, 96)

**6.** “compare the clinical effects and tolerability profile of treatments with extrafine HFA-beclomethasone-formoterol (BDP-F) versus equipotent doses of nonextrafine ICS-LABA” were corrected as “compare the clinical effects and tolerability profile of treatments with extrafine HFA-BDP-F versus equipotent doses of nonextrafine ICS-LABA”(Line 105)

**7.** “a significant decrease in FeNO levels were observed in extrafine BDP-F” were corrected as “a significant decrease in fractional exhaled nitric oxide (FeNO) levels were observed in extrafine BDP-F”(Line 95, 96)

**8.** “Airway inflammation: fractional exhaled nitric oxide (FeNO, ppb) and alveolar nitric oxide (C_Alv_, ppb); Asthma control: Asthma Control Test score” were corrected as “Airway inflammation: FeNO (ppb) and alveolar nitric oxide (C_Alv_, ppb); Asthma control: Asthma Control Test (ACT) score”(Line 130, 131)

**9.** “Patients were randomized to either extrafine HFA-beclomethasone/formoterol (BDP-F) via pMDI (FOSTER^®^; Chiesi Farmaceutici, Italy) [18-25, 30, 31] or nonextrafine ICS-LABA, including fluticasone/salmeterol (FP/S) via DPI (Seretide^®^ Diskus^®^; GlaxoSmithKline, UK)[18-20, 23, 31], FP/S via pMDI (Seretide^®^; GlaxoSmithKline, UK) [21, 24], budesonide/formoterol (BUD/F) via DPI (Symbicort^®^ Turbuhaler^®^; AstraZeneca, Sweden) [20, 22, 25] and CFC-BDP” were corrected as “Patients were randomized to either extrafine HFA-BDP-F via pMDI (FOSTER^®^; Chiesi Farmaceutici, Italy) [18-25, 31, 32] or nonextrafine ICS-LABA, including FP-S via DPI (Seretide^®^ Diskus^®^; GlaxoSmithKline, UK)[18-20, 23, 32], FP/S via pMDI (Seretide^®^; GlaxoSmithKline, UK) [21, 24], BUD-F via DPI (Symbicort^®^ Turbuhaler^®^; AstraZeneca, Sweden) [20, 22, 25] and Chloroflurocarbon (CFC)-BDP ”(Line 195-200)

**10.** “laboratory parameters and ECG” were corrected as “laboratory parameters and electrocardiogram (ECG)”(Line 288)

**11.** “an elevation of RV or RV/TLC ratio” were corrected as “an elevation of residual volume (RV) or RV/total lung capacity (TLC) ratio ”(Line 326, 327)

**12.** We have add one reference. (Line 453-455)

We hope that the revised version of the manuscript is now acceptable for publication in your journal. If there is anything else we should do, please do not hesitate to let us know.

I look forward to hearing from you soon.

Thank you very much.

Yours sincerely,

Chuntao Liu
